# Serotonergic Suppression of Sustained Synaptic Responses in Rat Oculomotor Neural Integrator Networks

**DOI:** 10.1523/ENEURO.0352-25.2025

**Published:** 2025-12-12

**Authors:** Yasuhiko Saito, Taketoshi Sugimura

**Affiliations:** Department of Neurophysiology, Nara Medical University, Kashihara 634-8521, Japan

**Keywords:** excitatory network, gaze holding, neural integrator, serotonin, slice, whole-cell recording

## Abstract

Neural signals necessary for gaze holding are produced in the excitatory networks of oculomotor neural integrators including the prepositus hypoglossi nucleus (PHN) and the interstitial nucleus of Cajal (INC). Our previous studies have shown that the activation of the networks can be evaluated by sustained excitatory postsynaptic current (EPSC) responses in vitro, in which a higher EPSC frequency after burst stimulation (100 Hz, 20 trains) than the frequency before the stimulation lasts for >1 s. Both the PHN and the INC receive serotonergic inputs mainly from the dorsal raphe nucleus, and serotonin (5-HT) induces depolarizing responses via 5-HT_2_ or 5-HT_3_ receptors and hyperpolarizing responses via 5-HT_1A_ receptors in PHN and INC neurons. However, how 5-HT affects sustained EPSC responses remains unknown. In this study, we investigated the effects of 5-HT on sustained EPSC responses using whole-cell recordings in brainstem slices obtained from rats of either sex. Compared with the control treatment, bath application of 10 μM 5-HT significantly reduced the duration and frequency of the EPSC responses in the PHN and the INC. The application of 8-OH-DPAT, an agonist of the 5-HT_1A_ receptor, suppressed sustained EPSC responses, but agonists of the 5-HT_2_ and 5-HT_3_ receptors had no effect on the responses, indicating that 5-HT has a suppressive effect on sustained EPSC responses via 5-HT_1A_ receptors. These results suggest that neurons that express 5-HT_1A_ receptors participate in excitatory networks and that the suppressive effect of 5-HT can facilitate exploratory behavior through eye movements rather than gaze holding.

## Significance Statement

Excitatory networks of brainstem oculomotor neural integrators are involved in gaze holding. Although neural integrators receive serotonergic inputs, how serotonergic inputs modulate the activity of excitatory networks remains unknown. We investigated the effect of serotonin (5-HT) on network activity using whole-cell recordings in brainstem slices. The finding that 5-HT suppressed network activity via 5-HT_1A_ receptors but not via 5-HT_2_ and 5-HT_3_ receptors indicates that 5-HT suppresses network activity via 5-HT_1A_ receptors. This finding suggests that the neurons that express 5-HT_1A_ participate in the excitatory networks of oculomotor neural integrators and that the suppressive effect of 5-HT can facilitate exploratory behavior through eye movements rather than gaze holding.

## Introduction

Oculomotor neural integrators, which convert transient signals to sustained signals, are involved in gaze holding, which is executed by the tonic contraction of extraocular muscles. Neural networks, including the prepositus hypoglossi nucleus (PHN) and interstitial nucleus of Cajal (INC), are known to be neural integrators that participate in horizontal and vertical gaze, respectively (Skavenski and Robinson, 1973; Robinson, 1975, 1989; Fukushima, 1987, 1991; Fukushima et al., 1992; Fukushima and Kaneko, 1995; Leigh and Zee, 2015). One of the mechanisms through which sustained activity is achieved to enable tonic contraction may be the sustained activation of excitatory networks, including the PHN and the INC. Our previous in vitro studies have demonstrated that under a blockade of inhibitory synaptic transmission, the application of burst stimulation (100 Hz, 0.2 s) to a nearby site of a recorded neuron in the PHN or the INC induced an increase in the frequency of spontaneous excitatory postsynaptic currents (EPSCs) that lasted for several seconds (Saito and Yanagawa, 2010; Saito et al., 2017; Saito and Sugimura, 2020). In addition, analyses using various stimulation parameters have revealed that sustained EPSC responses are indicative of excitatory network activation. Moreover, pharmacological analyses have shown that sustained excitatory network activation through PHN is mediated predominantly via Ca^2+^-permeable (CP) α-amino-3-hydroxy-5-methyl-4-isoxazolepropionic acid (AMPA) receptors and Ca^2+^-activated nonselective cation channels, whereas its activation through the INC is mediated predominantly via *N*-methyl-d-aspartate (NMDA) receptors (Saito and Sugimura, 2020).

The PHN receives serotonergic innervations that originate primarily from the dorsal raphe nucleus (Vertes and Kocsis, 1994; Cuccurazzu and Halberstadt, 2008). Electrophysiological studies in slice preparations have shown that PHN neurons become hyperpolarized via 5-HT_1A_ receptors and depolarized via 5-HT_2_ receptors in response to bath application of 5-HT (Bobker and Williams, 1989, 1990, 1995; Bobker, 1994; Idoux et al., 2006). Our recent study using local puff application of 5-HT revealed three types of 5-HT–induced current responses in both PHN and INC neurons: 5-HT_3_ receptor-mediated fast inward current responses, 5-HT_1A_ receptor-mediated slow outward current responses, and 5-HT_2_ receptor-mediated slow inward current responses (Saito and Sugimura, 2023). Although these findings suggest that the activities of PHN and INC neurons are indeed modulated by 5-HT, how 5-HT affects the activity of excitatory networks, including those involving the PHN and the INC, remains unknown. Therefore, in this study, we investigated the effects of 5-HT and agonists of 5-HT receptor subtypes on sustained EPSC responses using whole-cell current recordings in rat brainstem slices.

## Materials and Methods

### Experimental animals

All experimental procedures were approved by the Animal Care Committee of Nara Medical University, and the experiments were carried out in accordance with the guidelines outlined by the US National Institutes of Health regarding the care and use of animals for experimental research (ARRIVE guidelines; Percie du Sert et al., 2020). Every effort was made to minimize the number of animals used and their suffering. In this study, 27 wild-type rats (Postnatal Days 18–21) of either sex were used for experiments.

### Slice preparation and whole-cell recording

The slice preparation and whole-cell patch–clamp recording procedures were similar to previously described procedures (Saito and Yanagawa, 2010; Saito et al., 2017; Saito and Sugimura, 2020). Briefly, the rats were deeply anesthetized with isoflurane and decapitated. The adequacy of the depth of anesthesia was judged by the absence of reflex movements to toe pinches. Frontal brain slices (400 μm thick for EPSC responses and 250 μm thick for current responses), which included the PHN or the INC, were cut using a microslicer (Pro 7, Dosaka) in an ice-cold sucrose solution containing (in mM) 234 sucrose, 2.5 KCl, 1.25 NaH_2_PO_4_, 10 MgSO_4_, 0.5 CaCl_2_, 26 NaHCO_3_, and 11 glucose, bubbled with 95% O_2_ and 5% CO_2_. The slices were recovered in an interface-type chamber perfused with an extracellular solution containing (in mM) 125 NaCl, 2.5 KCl, 2 CaCl_2_, 1 MgCl_2_, 1.25 NaH_2_PO_4_, 26 NaHCO_3_, and 25 glucose and aerated with 95% O_2_ and 5% CO_2_, pH 7.4, at 33°C for 1 h. After recovery, the slices were incubated in an aerated extracellular solution at room temperature. For recordings, each slice was placed in a submerged recording chamber on an upright microscope (Leica DM LFS, Leica Microsystems) and continuously perfused with the extracellular solution at a rate of 3 ml/min. The bath temperature was maintained at 30–32°C using an in-line heater (SH-27A; Warner Instruments). Whole-cell current recordings were performed from healthy-looking neurons within the PHN and the INC via Nomarski microscopy using an EPC-8 patch-clamp amplifier (HEKA), and the data were acquired using a pClamp9 system (Molecular Devices). Patch pipettes were prepared from borosilicate glass capillaries and filled with either of the following internal solutions: a Cs^+^-based internal solution that contained (in mM) 145 Cs-gluconate, 5 CsCl, 0.2 EGTA, 2 MgATP, 0.3 NaGTP, 10 HEPES, 0.1 spermine, and 5 lidocaine *N*-ethyl bromide (QX-314) or a K^+^-based internal solution that contained (in mM) 120 K-methylsulfate, 10 KCl, 0.2 EGTA, 2 MgATP, 0.3 NaGTP, 10 HEPES, 10 Na_2_-phosphocreatine, and 0.1 spermine. The pH of the Cs^+^-based and K^+^-based internal solutions was adjusted to 7.3 with gluconate and KOH, respectively. The osmolarity of the internal solution ranged from 280 to 290 mOsm/L, and the resistance of the patch electrodes ranged from 5 to 9 MΩ in the bath solution. The Cs^+^-based internal solution was used for recording EPSC responses and current responses induced by a local application of glutamate receptor agonists, and the K^+^-based internal solution was used for current responses induced by 5-HT and its agonist. The membrane potential of a recorded neuron was held at −75 mV during the recordings. In EPSC responses, burst stimulation with 20 cathodal square-wave pulses (50–60 μA, 100 μs in duration) was constantly applied during the experiment at 40 s intervals near a recorded neuron using a glass micropipette that was filled with the extracellular solution. The site where the current response of the neuron was the greatest was determined as an appropriate stimulation site. EPSC responses were recorded in an extracellular solution containing 20 μM strychnine and 100 μM picrotoxin (control solution). The control solution containing 5-HT or the agonists of 5-HT receptor subtypes was bath-applied for 5 min, after which the control solution was applied to wash out the agonists. The 5-HT receptors exhibit different affinities for 5-HT depending on their subtypes; 5-HT1 receptors exhibit nanomolar affinities for 5-HT, whereas 5-HT2 and 5-HT3 receptors exhibit micromolar affinities for 5-HT (van Hooft and Vijverberg, 2000; Marin et al., 2020; Barnes et al., 2021). In this study, the concentration of 5-HT (10 μM) was set on the basis of previous studies that demonstrated its efficacy (Andrade et al., 1986; Takahashi and Berger, 1990; Kjaerulff and Kiehn, 2001; Zhang, 2003; Villalobos et al., 2005). For current responses, 5-HT (1 mM), kainate (1 mM), or NMDA (1 mM) was applied to the soma of a recorded neuron via pressurized air (30 psi, 5 ms) using a pneumatic PicoPump (PV820; World Precision Instruments) at 40 s intervals. AMPA receptor-mediated current responses induced by kainate were recorded at holding potentials of −60 and +40 mV to determine the rectification index (RI), where RI > 1 and RI < 1 indicate Ca^2+^-impermeable and Ca^2+^-permeable, respectively (Ozawa et al., 1991; Saito and Yanagawa, 2010). NMDA receptor-mediated current responses were recorded at holding potentials of +40 mV to prevent Mg^2+^ blockage. Current signals were low-pass filtered at 1 kHz and digitized at 5 kHz. The measured liquid junction potentials of −10 mV for the Cs^+^-based internal solution and −5 mV for the K^+^-based internal solution were corrected. Neurons that displayed membrane potentials of less than −50 mV immediately following patch membrane rupture and that exhibited spontaneous EPSCs were used in the analyses.

### Drugs

The 5-HT and *N*-[2-[4-(2-methoxyphenyl)-1-piperazinyl]ethyl]-*N*-2-pyridinylcyclohexane-carboxamide (WAY-100635) maleate salt were purchased from Sigma-Aldrich, SR57227 hydrochloride and (±)-8-hydroxy-2-dipropylaminotetralin hydrobromide (8OH-DPAT) were purchased from Bio-Techne/R&D Systems, α-methyl-5-hydroxytryptamine maleate salt (α-methyl 5-HT) was purchased from Santa Cruz Biotechnology, and the other drugs were purchased from FUJIFILM Wako Pure Chemical. The 5-HT receptor-related drugs were dissolved in water supplemented with 100 μM sodium metabisulfite to prevent oxidation. Serotonin (5-HT) and SR57227 were stored at concentrations of 50 mM, and the other antagonists were stored at concentrations of 1,000 times the final concentration. The stock solutions were stored at −20°C before being diluted in the extracellular solution.

### Data analysis

Offline analysis was performed using the AxoGraph X software (AxoGraph Scientific). EPSCs were determined when the peak of the inward current was greater than three times the standard deviation (SD) of the baseline current before burst stimulation. We usually obtained five to six control recordings of sustained EPSC responses after burst stimulation and seven or eight recordings during the 5 min period of 5-HT administration. The EPSC response induced by burst stimulation was estimated by two parameters: the duration of the sustained EPSC response and the increase in EPSC frequency. To estimate the duration of the sustained EPSC response, we constructed a histogram showing the EPSC frequency against time. The histogram was constructed from the last three recordings before (control) and during 5-HT administration. Using the histogram, the duration was defined as the period from when the burst stimulation was terminated to when the average value of three adjacent bins (corresponding to 300 ms) became equal to or smaller than the average baseline EPSC frequency before the burst stimulation. The increase in EPSC frequency was measured from recordings taken 1 s after burst stimulation. The baseline EPSC frequency was measured from recordings taken 2 s before the burst stimulation. The RI was calculated using the formula RI = (*I*_+40_ / +40) / (*I*_−60_ / _−_60), where *I*_+40_ and *I*_−60_ represent the amplitudes of AMPA receptor-mediated currents at membrane potentials of +40 and −60 mV from the reversal potential, respectively. All values are reported as the mean ± SD. Data normality was determined using the Shapiro–Wilk test. Paired data were statistically analyzed using paired Student's *t* tests (normally distributed data) and Wilcoxon signed-rank tests (non-normally distributed data). Statistical significance was defined as *p* < 0.05. These analyses were performed using the StatView software (ver. 5.0; Hulinks) and JMP software (ver. 6.0.2). Post hoc power analysis was performed using the G*Power3 software (ver. 3.1.9.4, http://www.gpower.hhu.de/; Faul et al., 2007). The results of the statistical analyses are shown in Table 1.

**Table 1.** Statistical test

Label	Parameter (unit)	Bivariate	Cell#	Mean	SD	Distribution, *p* value (type of test)	Power (*α* = 0.05)
A1	Duration of PHN neurons (s)	Control	7	2.3	0.7	Normal, *p* = 0.0008 (Paired *t* test)	0.999
		5-HT	7	0.8	0.1		
A2	EPSC frequency of PHN neurons (event/s)	Control	7	39.2	12.3	Normal, *p* < 0.0001 (Paired *t* test)	1.000
		5-HT	7	20.1	12.4		
A3	Duration of INC neurons (s)	Control	8	2.9	1.4	Normal, *p* = 0.0129 (Paired *t* test)	0.907
		5-HT	8	1.2	0.4		
A4	EPSC frequency of INC neurons (event/s)	Control	8	21.6	7.6	Non-normal, *p* = 0.0117 (Wilcoxon signed-rank test)	0.997
		5-HT	8	15.6	5.5		
A5	Baseline EPSC frequency of PHN neurons (event/s)	Control	7	6.2	1.1	Normal, *p* = 0.942 (paired *t* test)	0.073
		5-HT	7	6.3	0.9		
A6	Baseline EPSC frequency of INC neurons (event/s)	Control	8	5.0	1.6	Normal, *p* = 0.493 (paired *t* test)	0.162
		5-HT	8	5.7	3.0		
B1	Duration of PHN neurons (s)	Control	6	2.7	1.1	Normal, *p* = 0.009 (paired *t* test)	0.970
		DPAT	6	1.0	0.3		
B2	EPSC frequency of PHN neurons (event/s)	Control	6	33.2	11.4	Normal, *p* = 0.008 (paired *t* test)	0.975
		DPAT	6	15.7	5.9		
B3	Duration of INC neurons (s)	Control	6	2.7	0.8	Normal, *p* = 0.004 (paired *t* test)	0.995
		DPAT	6	1.0	0.5		
B4	EPSC frequency of INC neurons (event/s)	Control	6	27.1	5.8	Non-normal, *p* = 0.028 (Wilcoxon signed-rank test)	1.000
		DPAT	6	13.7	3.4		
B5	Baseline EPSC frequency of PHN neurons (event/s)	Control	6	5.0	1.0	Normal, *p* = 0.506 (paired *t* test)	0.154
		DPAT	6	5.3	1.6		
B6	Baseline EPSC frequency of INC neurons (event/s)	Control	6	6.7	1.9	Normal, *p* = 0.065 (paired *t* test)	0.651
		DPAT	6	5.4	1.0		
C1	Duration of PHN neurons (s)	Control	6	2.1	0.3	Normal, *p* = 0.428 (paired *t* test)	0.184
		WAY	6	2.0	0.5		
C2	EPSC frequency of PHN neurons (event/s)	Control	6	26.3	5.5	Normal, *p* = 0.264 (paired *t* test)	0.289
		WAY	6	22.7	4.7		
C3	Duration of INC neurons (s)	Control	4	2.6	1.1	Normal, *p* = 0.907 (paired *t* test)	0.061
		WAY	4	2.6	1.2		
C4	EPSC frequency of INC neurons (event/s)	Control	4	31.9	6.3	Normal, *p* = 0.0803 (paired *t* test)	0.634
		WAY	4	24.6	2.4		
C5	Baseline EPSC frequency of PHN neurons (event/s)	Control	6	4.7	4.1	Non-normal, *p* = 0.345 (Wilcoxon signed-rank test)	0.088
		WAY	6	4.9	3.1		
C6	Baseline EPSC frequency of INC neurons (event/s)	Control	4	6.9	4.1	Normal, *p* = 0.591 (Paired *t* test)	0.121
		WAY	4	6.8	4.5		
D1	Duration of PHN neurons (s)	Control	6	2.6	1.0	Normal, *p* = 0.932 (Paired *t* test)	0.059
		α-Met	6	2.7	0.7		
D2	EPSC frequency of PHN neurons (event/s)	Control	6	29.2	12.2	Normal, *p* = 0.0143 (Paired *t* test)	0.844
		α-Met	6	27.5	11.6		
D3	Duration of INC neurons (s)	Control	6	2.7	1.2	Non-normal, *p* = 0.581 (Wilcoxon signed-rank test)	0.150
		α-Met	6	2.8	1.3		
D4	EPSC frequency of INC neurons (event/s)	Control	6	39.5	17.3	Normal, *p* = 0.088 (paired *t* test)	0.572
		α-Met	6	34.9	17.7		
D5	Baseline EPSC frequency of PHN neurons (event/s)	Control	6	3.7	1.3	Normal, *p* = 0.211 (paired *t* test)	0.347
		α-Met	6	4.4	1.5		
D6	Baseline EPSC frequency of INC neurons (event/s)	Control	6	6.7	4.5	Normal, *p* = 0.375 (paired *t* test)	0.213
		α-Met	6	5.8	3.4		
E1	Duration of PHN neurons (s)	Control	6	2.4	0.5	Normal, *p* = 0.175 (paired *t* test)	0.390
		SR	6	2.2	0.6		
E2	EPSC frequency of PHN neurons (event/s)	Control	6	53.3	10.0	Normal, *p* = 0.180 (paired *t* test)	0.390
		SR	6	48.0	9.5		
E3	Duration of INC neurons (s)	Control	6	2.5	0.5	Normal, *p* = 0.205 (paired *t* test)	0.350
		SR	6	2.1	0.4		
E4	EPSC frequency of INC neurons (event/s)	Control	6	43.3	11.8	Normal, *p* = 0.993 (paired *t* test)	0.051
		SR	6	43.3	9.4		
E5	Baseline EPSC frequency of PHN neurons (event/s)	Control	6	5.6	1.1	Normal, *p* = 0.368 (paired *t* test)	0.216
		SR	6	6.2	2.1		
E6	Baseline EPSC frequency of INC neurons (event/s)	Control	6	4.8	2.6	Normal, *p* = 0.341 (paired *t* test)	0.234
		SR	6	5.1	2.6		
F1	AMPA current density (A/F) RI < 1	Control	12	43.1	14.9	Non-normal, *p* = <0.0001 (Wilcoxon signed-rank test)	1.000
		5-HT	12	38.5	12.9		
F2	AMPA current density (A/F) RI ≥ 1	Control	6	35.7	16.6	Normal, *p* = 0.745 (paired *t* test)	0.088
		5-HT	6	36.0	15.9		
F3	NMDA current density (A/F)	Control	7	16.8	7.8	Normal, *p* = 0.510 (paired *t* test)	0.178
		5-HT	7	16.0	5.7		

## Results

**Figure 1.**
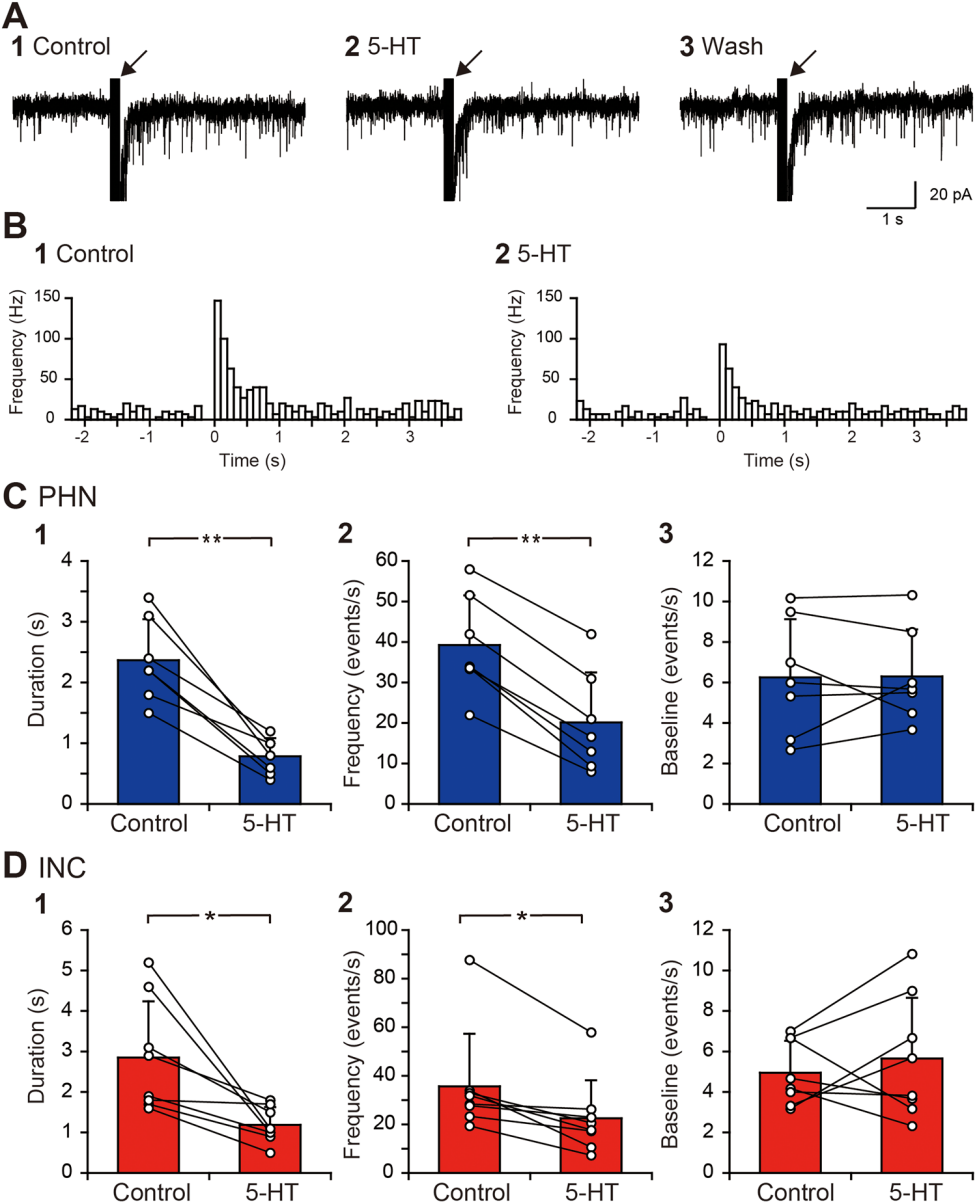
Decreases in the sustained EPSC response duration and EPSC frequency by serotonin. ***A***, Current responses of a PHN neuron to local burst stimulation in control solution (**1**), in solution containing 10 μM serotonin (5-HT; **2**), and after washing out of 5-HT (**3**). The arrow indicates the artifact induced by the burst stimulation. ***B***, Histograms showing the average EPSC frequency against time in the control (**1**) and in the presence of 5-HT (**2**). The width of the histogram bins is 100 ms. ***C***, ***D***, Comparisons of the duration of the increased EPSC frequency (**1**), the EPSC frequency for 1 s after burst stimulation (**2**), and the baseline EPSC frequency (**3**) of PHN (***C***) and INC (***D***) neurons before and during 5-HT application. *p < 0.05; **p < 0.01.

As in our previous studies (Saito and Yanagawa, 2010; Saito et al., 2017; Saito and Sugimura, 2020), when inhibitory synaptic transmission was blocked using 20 μM strychnine, an antagonist of glycine receptors, and 100 μM picrotoxin, an antagonist of GABA_A_ receptors, the application of burst stimulation near a recorded neuron within the PHN or the INC induced an increase in the EPSC frequency that lasted for several seconds, as exemplified in Figure 1*A*1. The EPSC frequency after burst stimulation was reduced by bath application of 10 μM 5-HT for 5 min (Fig. 1*A*2) and recovered from the reduction after >10 min of washing to remove 5-HT (Fig. 1*A*3). The histograms show the decrease in EPSC frequency caused by 5-HT not only immediately after burst stimulation but also after >1 s (Fig. 1*B*). The duration of the sustained EPSC response and the increase in EPSC frequency in the presence of 5-HT were significantly shorter and smaller than those in the control in PHN (Fig. 1*C*1,2; Table 1, A1, A2) and INC neurons (Fig. 1*D*1,2; Table 1, A3, A4). These results indicate that 5-HT suppressed the sustained EPSC response. Comparisons of the baseline EPSC frequency before burst stimulation revealed no significant difference between the control and 5-HT groups (Fig. 1*C*3; Table 1, A5; Fig. 1*D*3; Table 1, A6). In addition, the baseline EPSC amplitudes before and after 5-HT application were compared in individual neurons. Among the seven PHN neurons recorded, the EPSC amplitude of two neurons significantly increased after 5-HT application, whereas that of the remaining neurons did not change. Among the eight INC neurons recorded, one neuron showed a significant increase in EPSC amplitude, four neurons showed a significant decrease in the amplitude, and three neurons showed no change.

**Figure 2.**
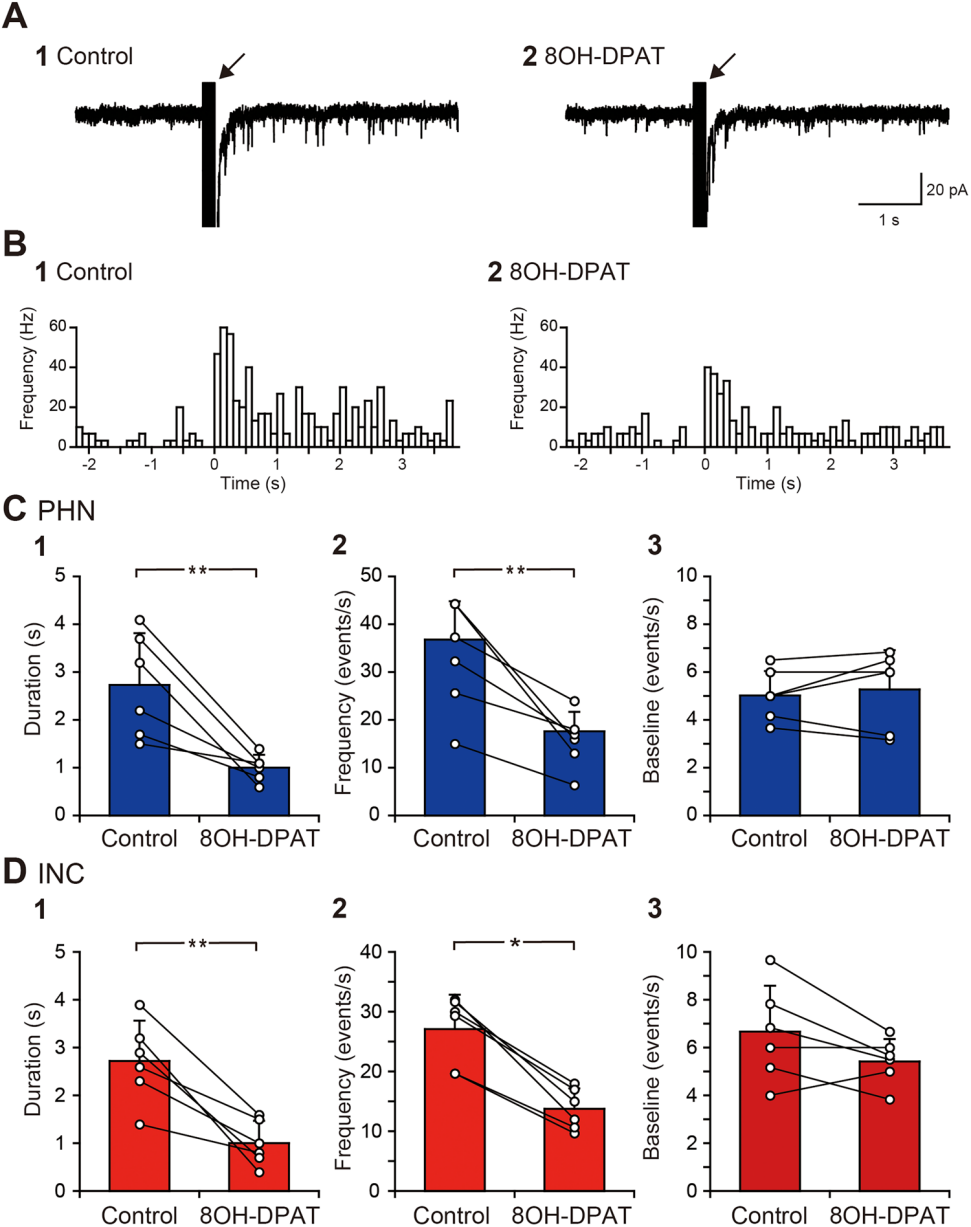
Decreases in the EPSC response duration and EPSC frequency by a 5-HT_1A_ receptor agonist. ***A***, Current responses of an INC neuron to local burst stimulation (arrow) in control solution (**1**) and in a solution containing 10 μM 8OH-DPAT (**2**). ***B***, Histograms (100 ms bin width) showing the averaged EPSC frequency against time in the control (**1**) and in the presence of 8OH-DPAT (**2**). ***C***, ***D***, Comparisons of the duration of increased EPSC frequency (**1**), EPSC frequency for 1 s after burst stimulation (**2**), and baseline EPSC frequency (**3**) of PHN (***C***) and INC (***D***) neurons before and during 8OH-DPAT application. *p < 0.05; **p < 0.01.

**Figure 3.**
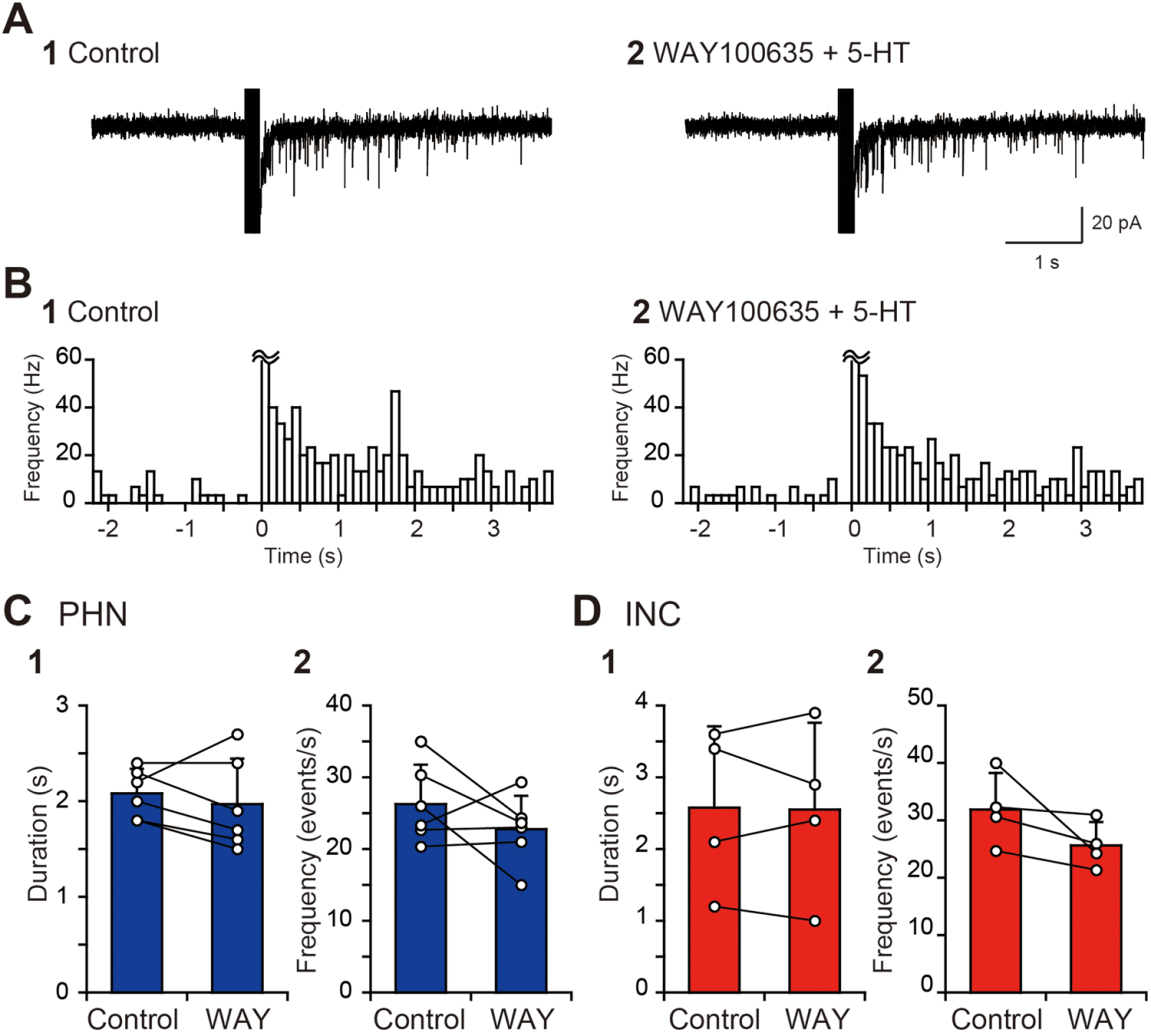
Blocking the effect of 5-HT on sustained EPSC responses by a 5-HT_1A_ receptor antagonist. ***A***, Current responses of an INC neuron to local burst stimulation (arrow) in control solution (**1**) and in a solution containing 10 μM 5-HT and 1 μM WAY100635 (**2**). ***B***, Histograms (100 ms bin width) showing the average EPSC frequency against time in the control (**1**) and in the presence of 5-HT and WAY100635 (**2**). ***C***, ***D***, Comparisons of the duration of the increased EPSC frequency (**1**) and the EPSC frequency for 1 s after burst stimulation (**2**) of PHN (***C***) and INC (***D***) neurons before and during 5-HT and WAY100635 application.

Our previous study demonstrated that 5-HT induces slow outward currents via 5-HT_1A_ receptors, slow inward currents via 5-HT_2_ receptors, and fast inward currents via 5-HT_3_ receptors in PHN and INC neurons (Saito and Sugimura, 2023). Therefore, 5-HT can both increase and decrease the activity of neural networks comprising PHN and INC neurons. Nevertheless, the suppression of the sustained EPSC response by 5-HT suggests that outward currents mediated by 5-HT_1A_ receptors predominantly affect the EPSC response. To test this possibility, we investigated the effect of 8OH-DPAT, an agonist of 5-HT_1A_ receptors, on the sustained EPSC response. The bath application of 10 μM 8OH-DPAT reduced the EPSC frequency, especially after >1 s (Fig. 2*A*,*B*). Comparisons of the duration of the sustained EPSC response and the increase in EPSC frequency revealed that the EPSC response duration and EPSC frequency after 8OH-DPAT application were significantly decreased in PHN (Fig. 2*C*1,2; Table 1, B1, B2) and INC (Fig. 2*D*1,2; Table 1, B3, B4) neurons. Similar to the results obtained with 5-HT application, the baseline EPSC frequency did not significantly differ between the control and the application of 8OH-DPAT in PHN (Fig. 2*C*3; Table 1, B5) and INC neurons (Fig. 2*D*3; Table 1, B6). To further confirm the involvement of 5-HT_1A_ receptors in the suppression of the sustained EPSC response, we investigated the effect of WAY100635, an antagonist of 5-HT_1A_ receptors, on 5-HT–induced suppression. The application of 10 μM 5-HT and 1 μM WAY100635 following the application of WAY100635 alone for 2 min did not obviously reduce the EPSC frequency (Fig. 3*A*,*B*). Comparisons of the duration of the sustained EPSC response and the increase in EPSC frequency revealed that the duration and frequency during 5-HT and WAY100635 application were not significantly different from those in the control in PHN (Fig. 3*C*1,2; Table 1, C1, C2) and INC neurons (Fig. 3*D*1,2; Table 1, C3, C4). The baseline EPSC frequency did not significantly differ between the control and the application of 5-HT and WAY100635 in PHN (Table 1, C5) and INC neurons (Table 1, C6). These results indicate that the suppressive effect of 5-HT on the sustained EPSC response is mediated by 5-HT_1A_ receptors.

**Figure 4.**
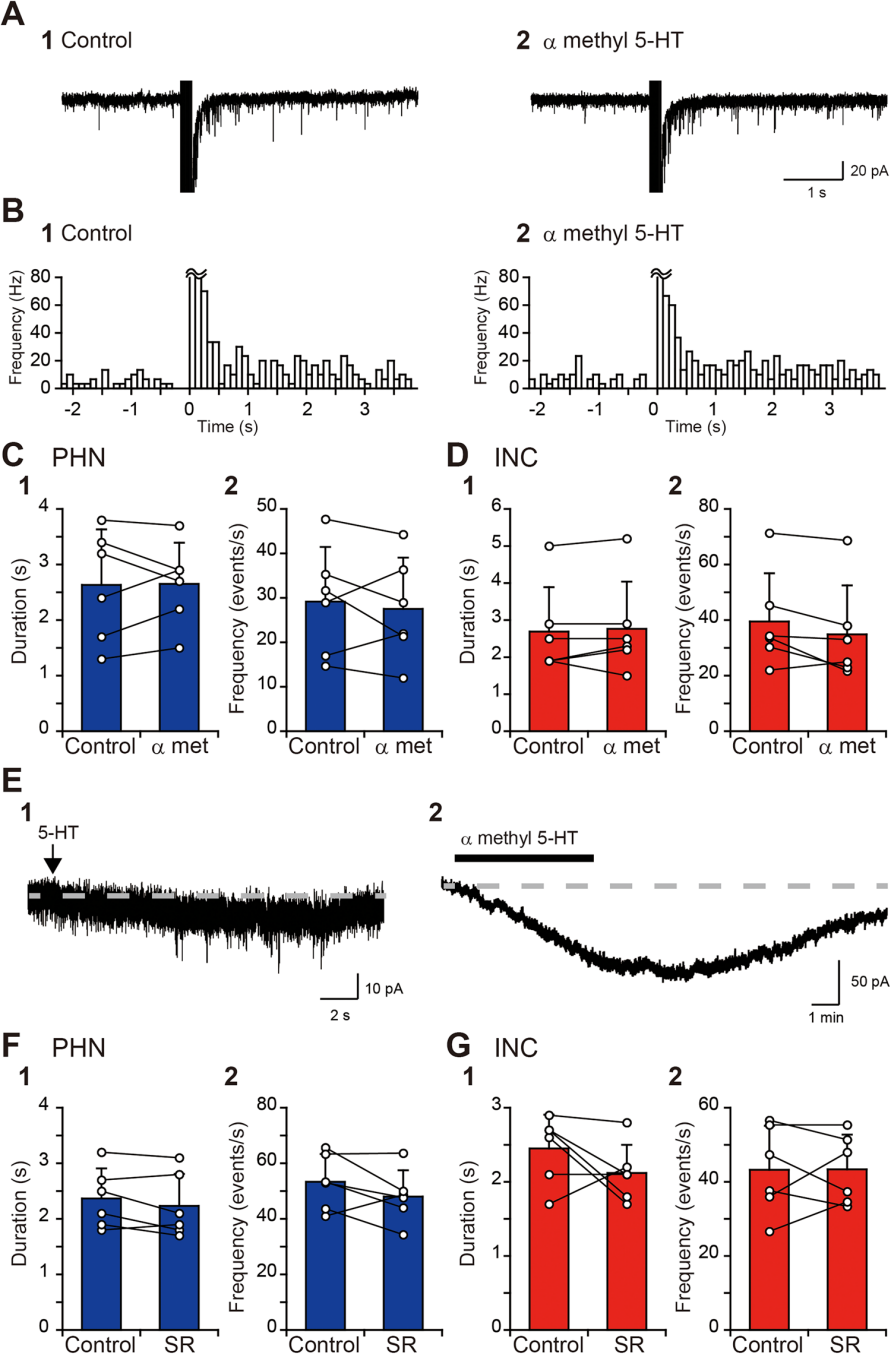
No significant effects of agonists of 5-HT_2_ and 5-HT_3_ receptors on sustained EPSC responses. ***A***, Current responses of a PHN neuron to local burst stimulation (arrow) in control solution (**1**) and in a solution containing 10 μM α-methyl 5-HT (**2**). ***B***, Histograms (100 ms bin width) showing the average EPSC frequency against time in the control (**1**) and in the presence of 5-HT and α-methyl 5-HT (**2**). ***C***, ***D***, Comparisons of the duration of the increased EPSC frequency (**1**) and the EPSC frequency for 1 s after burst stimulation (**2**) of PHN (***C***) and INC (***D***) neurons before and during α-methyl 5-HT application. ***E***, Inward currents induced by local application of 1 mM 5-HT (**1**) and a bath application of α-methyl 5-HT (**2**). The gray dashed line indicates an approximate baseline. ***F***, ***G***, Comparisons of the duration of the increased EPSC frequency (**1**) and the EPSC frequency for 1 s after burst stimulation (**2**) of PHN (***F***) and INC (***G***) neurons before and during 5 μM SR57227 application.

Although the sustained EPSC response in the PHN and INC is modulated by the activation of 5-HT_1A_ receptors, there are PHN and INC neurons that express 5-HT_2_ or 5-HT_3_ receptors (Saito and Sugimura, 2023). Therefore, we next investigated whether the activation of these receptors affects the sustained EPSC response. When 10 μM α-methyl 5-HT, an agonist of 5-HT_2_ receptors, was applied, the EPSC frequency after burst stimulation did not seem to change (Fig. 4*A*,*B*). Comparisons of the duration of the sustained EPSC response and the increase in EPSC frequency revealed that the EPSC response duration and EPSC frequency during α-methyl 5-HT application were not significantly different from those in the control in PHN (Fig. 4*C*1,2; Table 1, D1, D2) and INC neurons (Fig. 4*D*1,2; Table 1, D3, D4). Additionally, the baseline EPSC frequency during α-methyl 5-HT application did not significantly differ from that in the control in PHN neurons (Table 1, D5) or INC neurons (Table 1, D6). To exclude the possibility that the bath application of 10 μM α-methyl 5-HT might not be sufficiently effective for inducing inward currents, we investigated the current response to α-methyl 5-HT. All four PHN neurons and three INC neurons that showed inward currents by local application of 1 mM 5-HT (Fig. 4*E*1) showed inward currents after the bath application of α-methyl 5-HT (Fig. 4*E*2). The average peak currents across the PHN and INC neurons were −78.7 ± 40.3 and −60.9 ± 11.1 pA, respectively. The result that inward currents were induced in both PHN and INC neurons through the activation of 5-HT_2_ receptors suggests that 5-HT_2_ receptors do not participate in the modulation of the sustained EPSC response. We further investigated the involvement of 5-HT_3_ receptors in the sustained EPSC response. Bath application of 5 μM SR57227, an agonist of 5-HT_3_ receptors, did not significantly affect the duration or EPSC frequency in PHN (Fig. 4*F*1,2; Table 1, E1, E2) or INC neurons (Fig. 4*G*1,2; Table 1, E3, E4). The baseline EPSC frequency in PHN neurons (Table 1, E5) and INC neurons (Table 1, E6) was not significantly different between the SR57227-treated group and the control group. These results also indicate that 5-HT_3_ receptors do not participate in the modulation of the sustained EPSC response.

**Figure 5.**
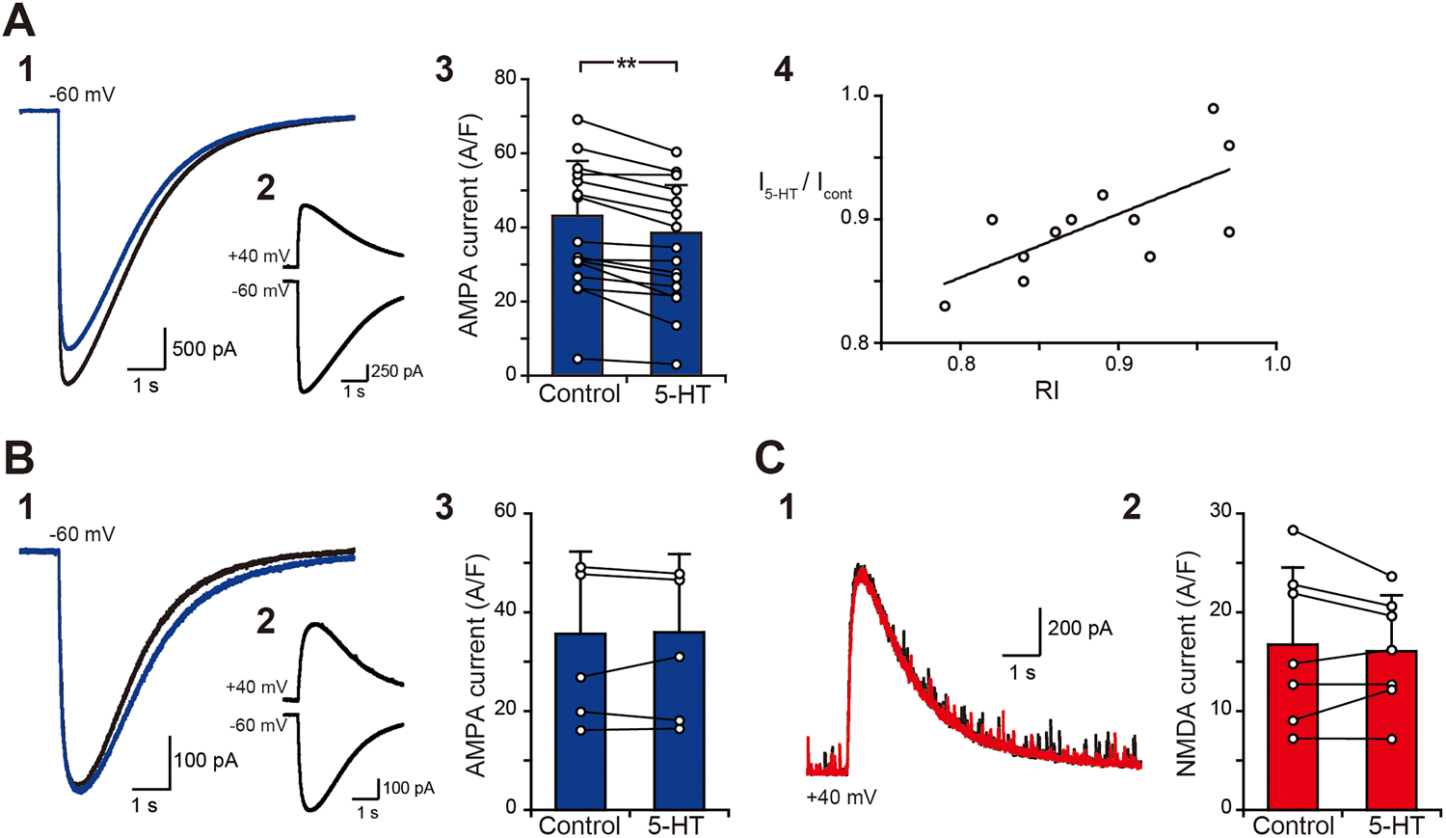
Effects of 5-HT on AMPA receptor- and NMDA receptor-mediated currents. ***A*1**, ***B*1**, Current traces of a PHN neuron to puff application of 1 mM kainate at a holding potential of −60 mV before (black) and after (blue) bath application of 10 μM 5-HT. ***A*2**, ***B*2**, Current responses of the PHN neuron to kainate at holding potentials of −60 and +40 mV. The RI values of the neurons in ***A*** and ***B*** are 0.84 and 1.18, respectively. ***A*3**, ***B*3**, Comparisons of the current density before and after the application of 5-HT in PHN neurons that showed RI < 1 (***A*3**, *n* = 12) and RI > 1 (***B*3**, *n* = 6). The plots connected by a line were obtained from individual PHN neurons. **p < 0.01. ***A*4**, Relationship between the RI and the ratio of the current in the control to the current in the presence of 5-HT in the 12 PHN neurons that showed RI < 1. ***C*1**, Current traces of an INC neuron to puff application of 1 mM NMDA at a holding potential of −40 mV before (black) and after (red) bath application of 10 μM 5-HT. ***C*2**, Comparison of the current density before and after the application of 5-HT to INC neurons.

Our previous studies indicated that sustained EPSC responses through the PHN are attributed mainly to the activation of CP-AMPA receptors, whereas EPSC responses through the INC are attributed to the activation of NMDA receptors (Saito and Sugimura, 2020). Therefore, the suppressive effect of 5-HT on sustained EPSC responses may suggest that the activation of CP-AMPA and NMDA receptors is modulated by 5-HT. To test this possibility, we recorded AMPA receptor-mediated currents in PHN neurons and NMDA receptor-mediated currents in INC neurons, which were induced by puff application of 1 mM kainate and 1 mM NMDA, respectively, and compared these currents before and after bath application of 5-HT. The duration of 5-HT application was 5 min because the effect of 5-HT on sustained EPSC responses was observed within 5 min. We analyzed 18 PHN neurons and 7 INC neurons, in which the change in series resistance was <5% before and after 5-HT application. Among the 18 PHN neurons, 12 neurons showed RI < 1 and 6 showed RI ≥ 1. The AMPA receptor-mediated currents before (black) and after (blue) bath application of 5-HT in PHN neurons that showed RI < 1 (Fig. 5*A*2) and RI ≥ 1 (Fig. 5*B*2) are shown in Figure 5, *A*1 and *B*1, respectively. Comparisons of the currents before and after 5-HT application revealed that the currents were significantly reduced in PHN neurons that showed RI < 1 (Fig. 5*A*3; Table 1, F1) but were not significantly different in PHN neurons that showed RI ≥ 1 (Fig. 5*B*3; Table 1, F2). In PHN neurons that showed RI < 1, the ratio of the AMPA receptor-mediated current after 5-HT application to the current before its application was positively correlated with the RI values (Fig. 5*A*4; *r* = 0.70; *p* = 0.0088). The NMDA receptor-mediated currents before (black) and after (red) the application of 5-HT to an INC neuron are shown in Figure 5*C*1. Comparisons of the current before and after 5-HT application revealed no significant difference in the current (Fig. 5*C*2; Table 1, F3). These results indicate that 5-HT reduces glutamatergic current responses in PHN neurons that showed RI < 1.

## Discussion

In this study, we investigated the effect of 5-HT on sustained EPSC responses in excitatory networks involving the PHN and the INC. The application of 5-HT reduced the EPSC response duration and EPSC frequency. However, the baseline EPSC frequency did not significantly change after 5-HT application and the change in the baseline EPSC amplitude was not consistent among the recorded neurons. These findings suggest that 5-HT does not have a consistent effect on the baseline EPSCs and rule out the possibility that the reduction in the sustained EPSC response is simply attributed to poor detection of EPSCs whose frequency is lower than the threshold of EPSC detection after 5-HT exposure. A reduction in the EPSC response was observed when a 5-HT_1A_ receptor agonist was applied but was not observed when 5-HT and a 5-HT_1A_ receptor antagonist were applied simultaneously. The application of 5-HT_2_ and 5-HT_3_ receptor agonists did not reduce the EPSC response duration or EPSC frequency. These results indicate that 5-HT has a suppressive effect on sustained EPSC responses via 5-HT_1A_ receptors.

The effect of 5-HT on the activity of integrator neurons has been intensively investigated in the PHN via intracellular recordings in guinea pig slice preparation (Bobker and Williams, 1989, 1990, 1995; Bobker, 1994; Idoux et al., 2006). According to these studies, PHN neurons are depolarized via 5-HT_2_ receptors and hyperpolarized via 5-HT_1A_ receptors. The depolarized and hyperpolarized responses may be attributed to the augmentation of hyperpolarization-activated currents (Ih) or the closure of K^+^ channels and the activation of G-protein–coupled inwardly rectifying K^+^ channels and/or Ca^2+^-dependent K^+^ channels, respectively (Bobker and Williams, 1989; Bobker, 1994). The induction of hyperpolarization and depolarization via 5-HT_1A_ and 5-HT_2_ receptors has also been demonstrated in other central neurons (Davies et al., 1987; Araneda and Andrade, 1991; Tanaka and North, 1993; Bengtson et al., 2004; Puig and Gulledge, 2011; Sengupta et al., 2017; Andrade and Haj-Dahmane, 2020). We previously confirmed 5-HT_1A_ receptor-mediated outward currents and 5-HT_2_ receptor-mediated inward currents both in PHN and INC neurons and further demonstrated that 5-HT_3_ receptors mediate fast inward currents (Saito and Sugimura, 2023). The results of the present study revealed that 5-HT reduced of the duration and frequency of EPSCs in response to burst stimulation. Although all 5-HT receptor subtypes should be activated by bath application of 5-HT, sustained EPSC responses were inhibited, and this effect was mediated by the 5-HT_1A_ receptor. These findings suggest that neurons that express 5-HT_1A_ receptors contribute to the sustained activation of excitatory networks involved in sustained EPSC responses but that neurons that express 5-HT_2_ or 5-HT_3_ receptors do not necessarily participate in the excitatory network. In addition to the postsynaptic effect of 5-HT, presynaptic modulation of synaptic transmission has been shown in several central synapses (Umemiya and Berger, 1995; Koyama et al., 1999; Blackmer et al., 2001; Bouryi and Lewis, 2003; Mizutani et al., 2006; Guo and Rainnie, 2010; Takenaka et al., 2011; Ding et al., 2013; Nishijo and Momiyama, 2016; Lu et al., 2018; Sato et al., 2023). Although we did not investigate the presynaptic effect of 5-HT on the sustained EPSC response in detail, the lack of a significant difference in the baseline EPSC frequency before and after 5-HT application suggests that the baseline network activity, including neurotransmitter release, may not greatly change even after 5-HT application.

The finding that 5-HT induces outward currents via 5-HT_1A_ receptors (Saito and Sugimura, 2023) indicates that 5-HT hyperpolarizes PHN and INC neurons to prevent the induction of sustained EPSC responses. In addition, we observed a significant reduction in AMPA receptor-mediated currents by 5-HT in PHN neurons that exhibited the RI < 1, although AMPA receptor-mediated currents did not significantly change in neurons that exhibited the RI > 1. These results suggest that 5-HT modulates CP-AMPA receptors but does not affect Ca^2+^-impermeable AMPA (CI-AMPA) receptors. Although we did not investigate whether the reduced currents induced by 5-HT were entirely mediated by CP-AMPA receptors, 5-HT can modulate the CP-AMPA receptor-mediated current response. The inhibitory effect of 5-HT_1A_ receptors on AMPA receptor-mediated currents has been reported in several types of central neurons (Cai et al., 2002; Costa et al., 2012; Yuen et al., 2014; Li et al., 2018; Nishijo et al., 2022), and generally, most glutamatergic currents are mediated by CI-AMPA receptors. Therefore, 5-HT may modify AMPA receptors differently in PHN neurons than in neurons in other brain areas. Taken together, these findings suggest that the hyperpolarizing effects of 5-HT_1A_ receptors and the modulation of CP-AMPA receptors may be the mechanisms underlying the serotonergic reduction in the activity of excitatory networks involving the PHN. Serotonin (5-HT) has also been reported to reduce NMDA receptor-mediated currents (Lopez-Garcia, 1998; Antri et al., 2008), but we did not observe a significant reduction in the current in INC neurons. Because local application of a high concentration of NMDA in normal external solution may saturate NMDA receptor-mediated currents, the inhibitory effect of 5-HT on the current response may be underestimated. Even if this is the case, the effect of the modulation of NMDA receptors may not be strong, and the hyperpolarizing effect via 5-HT_1A_ receptors, which may attenuate the activation of NMDA receptors during burst stimulation, seems to be a major mechanism underlying the serotonergic reduction of the activity of excitatory networks through the INC.

Because the activation of the 5-HT pathway alters various biological states, describing the simple effect of 5-HT on gaze control is difficult. In serotonin syndrome, which is caused by an excessive increase in serotonin transmission, ocular clonus (involuntary and irregular eye movement) may be observed (Ables and Nagubilli, 2010; Scotton et al., 2019). Jonassen et al. (2015) reported that compared with the placebo group, a group that took a selective serotonin reuptake inhibitor (SSRI), citalopram, had an increased saccade frequency and shorter fixation duration during face viewing. These findings suggest that the increase in 5-HT concentration induced by SSRIs results in frequent saccadic eye movement rather than eye fixation. Therefore, the suppressive effect of 5-HT on excitatory networks for gaze holding can facilitate exploratory behavior through eye movements.

## Synthesis

Reviewing Editor: David Schoppik, New York University Grossman School of Medicine Decisions are customarily a result of the Reviewing Editor and the peer reviewers coming together and discussing their recommendations until a consensus is reached. When revisions are invited, a fact-based synthesis statement explaining their decision and outlining what is needed to prepare a revision will be listed below. The following reviewer(s) agreed to reveal their identity: Fumihito Saitow. Note: If this manuscript was transferred from JNeurosci and a decision was made to accept the manuscript without peer review, a synthesis may not be available.

Major comments:

1. If 5-HT lowers the input resistance, I would expect both the current noise to increase and shunting inhibition to reduce EPSC amplitudes compared to control. This effect would make it harder for a threshold-based rule to detect valid, small-amplitude EPSCs, which could result in a lower average frequency even if the number of synaptic events before and after 5-HT application is the same. Did the authors monitor input resistance during the experiment to quantify how much, if any, shunting is affecting EPSC amplitude? Another check would be comparing the distribution of EPSC amplitudes and baseline current noise before and after 5-HT application to see if there is a change.

2. Regarding the detection of sustained EPSC responses, the authors only compared frequency, but are these EPSCs actually involved in action potential generation? If the authors carried out experiments with current-clamp recordings, please provide the data. In addition, statistics on EPSC amplitude should also be included in the table.

3. The authors only tested one serotonin concentration. Consider including a discussion of the relative affinities of each serotonin receptor (5-HT1A, 5-HT2, and 5-HT3 receptors) in the pathophysiological explanation.

Minor comments:

1. The methods do not include criteria for determining that recorded cells are in PHN and INC. This should be stated.

2. "...burst stimulation ... was applied at 40s intervals ..." Did burst stimulation begin as soon as 5-HT was applied? Did burst stimulation occur throughout the 5 minute epoch of 5-HT application? How long in seconds was the period used to compute average frequency reported in the histograms? I think the addition of a single panel showing a diagram of the experimental design would quickly help clear these sorts of questions for the reader.

3. To relate the results in this paper with prior work, I think it would help to know if the EPSC measurements are taken during the slow inward and slow outward currents. In other words, does serotonin both cause these slow currents and cause changes in response to burst stimulation or do these phenomena occur at different points in time? Aside from 4E, the presence of slow currents is not obvious in the examples. Is this because of the timescale shown?

4. "All four PHN neurons and three INC neurons that showed inward currents by local application of 1 mM 5-HT (Fig. 4E1) showed inward currents after the bath application of alpha-methyl 5-HT (Fig. 4E2, PHN:-78.7 +- 40.3 pA, INC: -60.9 +- 11.1 pA)" What do the values "PHN:-78.7 +- 40.3 pA, INC: -60.9 +- 11.1 pA" refer to? Peak current? Are the averages across cells or across trials for the cell shown in 4E2?

5. "To exclude the possibility that sufficient inward currents were not induced by the bath application of alpha-methyl 5- HT, we ..." This portion was hard to understand on first pass. Are you excluding the possibility that the dosage of alpha-methyl 5-HT is too low to have any effect on the cells by measuring inward current?

6. Fig. 4E: Please show the data before drug administration for longer.

7. Discussion: "receptor" is missing in multiple places. Please check carefully.

Example: "Although all 5-HT subtype should be" should be "Although all 5-HT receptor subtype should be".

8. Table: power of B3 statics

## Author Response

Major comments:

1. If 5-HT lowers the input resistance, I would expect both the current noise to increase and shunting inhibition to reduce EPSC amplitudes compared to control. This effect would make it harder for a threshold-based rule to detect valid, small-amplitude EPSCs, which could result in a lower average frequency even if the number of synaptic events before and after 5-HT application is the same. Did the authors monitor input resistance during the experiment to quantify how much, if any, shunting is affecting EPSC amplitude? Another check would be comparing the distribution of EPSC amplitudes and baseline current noise before and after 5-HT application to see if there is a change.

Reply: Because we did not measure the input resistance during EPSC recordings, we cannot estimate the effect of shunting on EPSCs. Following the reviewer's suggestion, we compared the amplitude of baseline EPSCs and the baseline noise (SD of the baseline current) before and after 5-HT application. Among the 7 PHN neurons recorded, the EPSC amplitude of two neurons significantly increased after 5-HT application, whereas that of the remaining neurons did not change. The baseline noise of four PHN neurons increased, whereas that of three PHN neurons decreased. Among the 8 INC neurons recorded, one neuron showed a significant increase in EPSC amplitude, four neurons showed a significant decrease in the amplitude, and three neurons showed no change. The baseline noise of three INC neurons increased, whereas that of five INC neurons decreased. Given that no consistent results were obtained, the shunting effect of 5-HT is not considered to have a significant effect on the baseline EPSCs. However, sustained EPSC responses were suppressed by 5-HT after burst stimulation. These findings suggest that the hyperpolarizing response induced by 5-HT attenuates the activation of CP-AMPA receptors and NMDA receptors during burst stimulation, thereby affecting only sustained EPSC responses. We have added this information to the text (Page 13, lines 3o9 of the first paragraph of Discussion; Page 16, lines 394 from the bottom of the first paragraph). Some readers may be concerned about changes in EPSC amplitude after 5-HT application; thus, we have also included information on the amplitude changes in the text (Page 9, lines 1o7 from the bottom of the first paragraph).

2. Regarding the detection of sustained EPSC responses, the authors only compared frequency, but are these EPSCs actually involved in action potential generation? If the authors carried out experiments with current-clamp recordings, please provide the data. In addition, statistics on EPSC amplitude should also be included in the table.

Reply: In our previous study (Saito and Yanagawa, 2010, J Neurosci 30:15735-15746), we performed current-clamp experiments in the PHN. After burst stimulation, the higher spike frequency than the frequency before the burst stimulation did not last more than 1 s in most PHN neurons. This result indicates that the synaptic events induced in this study have little effect on the sustained increase in the firing frequency. Excitatory networks that participate in neural integrators include not only the local network in the PHN and the INC but also networks with the cerebellum and vestibular nuclei and so on. Therefore, the small impact may be due to the difficulty in the induction of high numbers of synaptic events sufficient to cause a sustained increase in the firing frequency in slice preparations.

With respect to EPSC amplitude, comparisons before and after the 5-HT application were performed in individual neurons. As we have replied to the major comment 1, we have described the information on the amplitude changes in the text because we believe that incorporating these results into the table is not appropriate.

3. The authors only tested one serotonin concentration. Consider including a discussion of the relative affinities of each serotonin receptor (5-HT1A, 5-HT2, and 5-HT3 receptors) in the pathophysiological explanation.

Reply: 5-HT receptors exhibit different affinities for 5-HT depending on their subtypes; 5-HT1 receptors exhibit nanomolar affinities for 5-HT, whereas 5-HT2 and 5-HT3 receptors exhibit micromolar affinities for 5-HT (von Hooft and Vijverberg 2000; Marin et al. 2020; Barnes et al. 2021). As the reviewer suggested, the dose-dependence of 5-HT is an important point. However, because it was not easy to obtain successful recordings of sustained EPSC responses, it was challenging to investigate 5-HT dose-dependence. Therefore, the concentration of 5-HT was set on the basis of previous studies that demonstrated its efficacy without high concentrations (Andrade et al. 1986 Science; Takahashi and Berger 1990 JP; Kjaerulff and Kiehn 2001 JNP; Zhang 2003 JNS; Villalobos et al. 2005 EJN). We have added these comments to the text (Page 6, lines 6n12).

Minor comments:

1. The methods do not include criteria for determining that recorded cells are in PHN and INC. This should be stated.

Reply: When the data were recorded, no specific criteria were determined, particularly regarding the position of the neurons. Recordings were made from healthy-looking neurons within the PHN and the INC, and we applied burst stimulation near a recorded neuron that exhibited spontaneous EPSCs. We have added this description to the Methods section (Page 5, line 7; Page 6, the bottom lineRPage7, the first line).

2. "...burst stimulation ... was applied at 40s intervals ..." Did burst stimulation begin as soon as 5-HT was applied? Did burst stimulation occur throughout the 5 minute epoch of 5-HT application? How long in seconds was the period used to compute average frequency reported in the histograms? I think the addition of a single panel showing a diagram of the experimental design would quickly help clear these sorts of questions for the reader.

Reply: Burst stimulation was constantly delivered at 40-second intervals during the experiment. 5-HT was administered after the control recordings were completed (usually five to six recordings). Therefore, seven or eight recordings of sustained EPSC responses after burst stimulation were obtained during the 5-minute period of 5-HT administration. The histogram was constructed from the last three recordings before (control) and during 5-HT administration. It is quite difficult to make a diagram for this experiment, so I added the explanation described above to the text (Page 5, the bottom line; Page 7, lines 3e6 of the second paragraph; Page 8, lines 263).

3. To relate the results in this paper with prior work, I think it would help to know if the EPSC measurements are taken during the slow inward and slow outward currents. In other words, does serotonin both cause these slow currents and cause changes in response to burst stimulation or do these phenomena occur at different points in time? Aside from 4E, the presence of slow currents is not obvious in the examples. Is this because of the timescale shown? Reply: As the reviewer noted, slow inward or outward currents cannot be observed at this timescale when EPSC responses are recorded. However, on the basis of the reviewer's suggestion, we investigated changes in the baseline shift before and after 5-HT administration. Among the 7 PHN neurons recorded, one showed an outward shift (16.5 pA) in the baseline current, and the reminder showed an inward shift (18.9 R 23.5 pA ranged from 2.1 pA to 65.8 pA). Among the 8 INC neurons recorded, one showed an outward shift (0.7 pA) in the baseline current and the reminder showed an inward shift (21.7 i 20.5 pA ranged from 10.1 pA to 61.3 pA). These results may have occurred because many neurons that exhibit inward current responses to 5-HT were recorded in this study. Or there were some neurons that became leaky during recording. Regardless, the recorded neurons themselves must also have been affected by 5-HT. As in the response to major comment 1, the hyperpolarizing effect of 5-HT may attenuate the activation of CP-AMPA receptors and NMDA receptors during burst stimulation, thereby affecting sustained EPSC responses.

4. "All four PHN neurons and three INC neurons that showed inward currents by local application of 1 mM 5-HT (Fig. 4E1) showed inward currents after the bath application of alpha-methyl 5-HT (Fig. 4E2, PHN:-78.7 +- 40.3 pA, INC: -60.9 +- 11.1 pA)" What do the values "PHN:-78.7 +- 40.3 pA, INC: -60.9 +- 11.1 pA" refer to? Peak current? Are the averages across cells or across trials for the cell shown in 4E2? Reply: The values represent the average peak current across the cells. We have added this explanation to the text (Page 11, lines 6R7 from the bottom).

5. "To exclude the possibility that sufficient inward currents were not induced by the bath application of alpha-methyl 5- HT, we ..." This portion was hard to understand on first pass. Are you excluding the possibility that the dosage of alpha-methyl 5-HT is too low to have any effect on the cells by measuring inward current? Reply: As the reviewer noted, we were concerned that this concentration of alpha-methyl 5-HT might not be sufficiently effective to induce inward currents. To make this text easier to understand, we have modified the description as follows, "To exclude the possibility that the bath application of 10 RM alpha-methyl 5-HT might not be sufficiently effective for inducing inward currents", ... (Page 11, lines 12M14).

6. Fig. 4E: Please show the data before drug administration for longer.

Reply: Unfortunately, we did not perform a longer recording before 5-HT administration.

In this experiment, we applied 5-HT when the baseline current was quite stable during the first 30 seconds or so. The other traces were similar.

7. Discussion: "receptor" is missing in multiple places. Please check carefully.

Example: "Although all 5-HT subtype should be" should be "Although all 5-HT receptor subtype should be".

Reply: We appreciate the reviewer's suggestion. We have added the description to the text.

8. Table: power of B3 statics Reply: We have corrected this error (Table1, B3).
